# Characteristics of Surface Acoustic Wave Sensors with Nanoparticles Embedded in Polymer Sensitive Layers for VOC Detection

**DOI:** 10.3390/s18072401

**Published:** 2018-07-23

**Authors:** Cristian Viespe, Dana Miu

**Affiliations:** Laser Department, Plasma and Radiation Physics, National Institute for Laser, Atomistilor # 409, 077125 Bucharest-Magurele, Romania; cristian.viespe@inflpr.ro

**Keywords:** SAW sensor, polymer nanocomposite, nanoparticle, VOC detection, pulsed laser ablation

## Abstract

Surface Acoustic Wave (SAW) sensors with several types of polymer sensing films, containing embedded Fe_3_O_4_ nanoparticles (NPs) with various dimensions and concentrations, were studied. A sensor with a sensing film consisting of the polymer alone was used for comparison. NPs with a mean diameter of 7 nm were produced by laser ablation with 5 ns pulse durations, and NPs with 13 nm diameters were obtained with a laser having 10 ps pulse durations. The properties of the Surface Acoustic Wave sensors with such sensing films were analyzed. Their response (frequency shift, sensitivity, noise and response time) to three different volatile organic components (VOCs) at various concentrations were compared with one another. The frequency shift and sensitivity increased with increasing NP concentration in the polymer for a given NP dimension and with decreasing NP diameter for a given concentration. The best results were obtained for the smallest NPs used. The SAW sensor containing 7 nm NPs had a limit of detection (LOD) of 65 ppm (almost five times better than the sensor with polymer alone), and a response time of about 9 s for ethanol.

## 1. Introduction

Gas sensors have gained great importance in numerous domains, such as in the detection of Chemical Warfare Agents (CWAs), control of emissions, and monitoring of various gases [1]. Several types of gas sensors are currently used, such as resistive sensors and optical devices [2]. Compared to other types of sensors, Surface Acoustic Wave (SAW) sensors have important advantages, such as high sensitivity, fast response time, low cost, ease of fabrication, room temperature operation and/or the possibility of wireless operation [1,3]. Improvements of SAW sensor properties have been achieved by using nanoporous sensitive films [4,5] or various nanostructures, such as bilayers [6,7,8], incorporated nanowires, or nanotubes [9,10].

Among the materials used for the sensitive layers of SAW sensors are polymers. Polymers have high sensitivities and fast responses for various vapors and gases, including toxic gases [11,12,13]. In particular, polyethylenimine (PEI) has proven to have a good response to CWA [14,15]. In literature, numerous methods for polymer deposition onto the surface of SAW sensors have been described, such as Laser-Induced Forward Transfer (LIFT) [16,17], spin coating [18], spray coating [19], etc.

Polymers, however, have a series of disadvantages, such as high losses, low propagation velocity or instability due to affinity to moisture [20]. On the other hand, inorganic metal oxides, which are the other class of materials often used in SAW sensors, have high sensitivities only at relatively high temperatures, and do not operate efficiently at room temperature [21]. In recent years, considerable research effort has gone into hybrid nanocomposites based on incorporation of such inorganic nanomaterials into polymer matrices [21]. Hybrid nanocomposites have led to gas sensing properties which are improved in comparison to both polymers alone and to inorganic metal oxides [22]. Such sensor layers based on polymers have a variety of properties that are better than the base polymers, mainly due to their high specific surface area, large number of sites at which gases can react [10,23], and enhancement of the film conductivity [24]. Incorporation of nanoparticles (NPs) into the polymer sensitive layer, for example, significantly improves the sensitivity of the sensor [19]. Studies have also shown that NPs improve the uniformity of nanocomposite polymer films which contain them [25], and eliminate dewetting of the layer, which is extremely detrimental in SAW sensors [26].

The present paper describes the properties of SAW sensors with nanocomposite sensitive layers consisting in a polymer (PEI) containing embedded Fe_3_O_4_ nanoparticles with different diameters and in different concentrations. The sensors were tested on Volatile Organic Compounds (VOCs). The role of the NP dimensions and concentrations in determining essential sensor properties such as frequency shift, sensitivity, noise and response time were investigated. In particular, the influence of NP dimensions on SAW sensor properties has not, to our best knowledge, been investigated.

## 2. Materials and Methods

The SAW sensor, designed as a two-port resonator, was fabricated on a ST-X cut quartz substrate and has an operating frequency of ~69 MHz. The SAW transducers were fabricated using photolithography and have Interdigital Transducers (IDTs) with Cr and Au metallization film thicknesses of 10 and 150 nm, respectively. Each input and output interdigital transducers consisted of 50 electrode pairs, aperture width 2500 µm, and a wavelength of ~45 µm; the distance between IDTs was 10 mm.

SAW sensors with several types of sensing films were studied, as presented in Table 1: Polymer (polyethylenimine-PEI) nanocomposite films containing Fe_3_O_4_ nanoparticles having various dimensions (7 nm, 13 nm or 50 nm) with the same mass concentration (0.4 mg NPs/mL polymer solution), and polymer nanocomposite films having the same NP dimensions (50 nm) with various concentrations of NPs (0.2, 0.4 or 0.8 mg NPs/mL polymer solution). A sensor with a sensing film consisting of the polymer alone was used for comparison.

**Figure 1:**
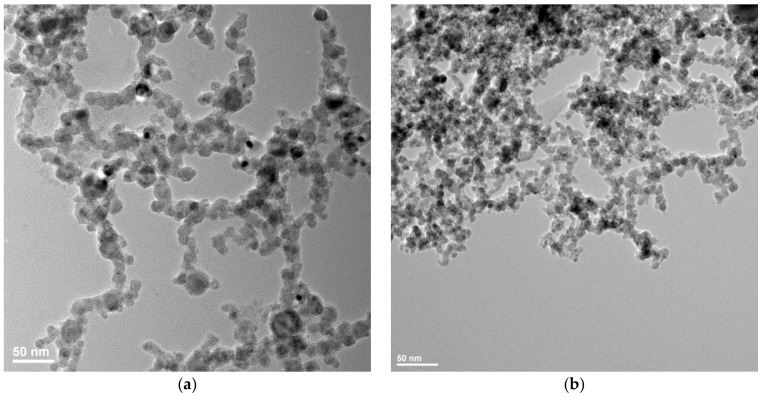
Transmission Electronic Microscopy (TEM) images of the nanoparticles obtained using (**a**) a picosecond laser and (**b**) a nanosecond laser, which are used in the nanocomposite Surface Acoustic Wave (SAW) sensitive layer (S2 and S3 respectively).

**Figure 2:**
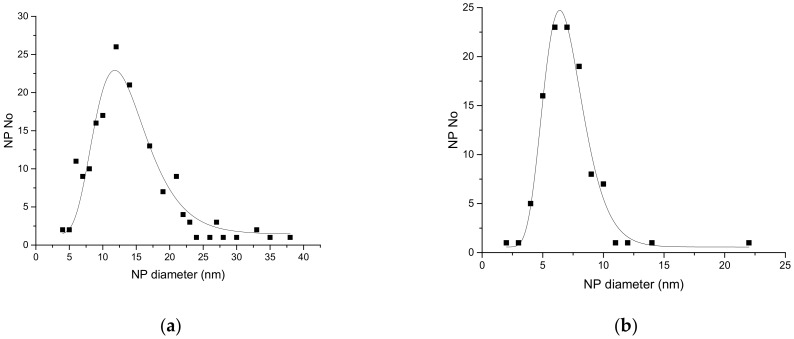
Distribution of nanoparticles (NP) diameters as obtained using TEM images in the case of (**a**) ps laser ablation and (**b**) ns laser ablation. Line is fit with a lognormal function.

**Figure 3:**
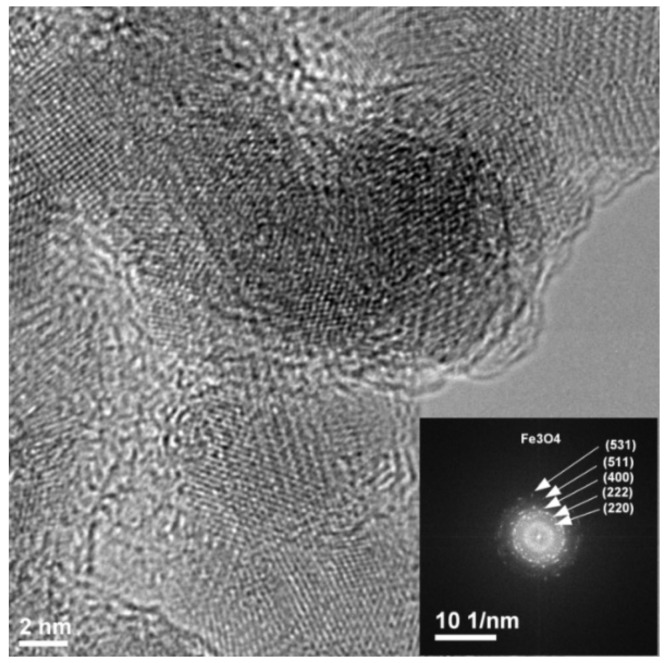
TEM image and SAED of a nanoparticle.

The NPs with a mean diameter of about 7 nm were produced by laser ablation using a Nd-YAG laser (EKSPLA NL301HT, EKSPLA uab, Vilnius, Lithuania) which had pulse durations of 5 ns and a repetition rate of 10 Hz, while those with about 13 nm diameters were obtained with a Nd-YVO_4_ laser (Lumera Rapid, Lumera Laser GmbH, Kaiserslautern, Germany) with 10 ps pulse durations and a repetition rate of 500 kHz. A laser wavelength of 355 nm was used in both cases to obtain the nanoparticles in an irradiation chamber with a controlled He atmosphere. A Fe target was irradiated in 400 Torr He, and the resulting nanoparticles were collected on a membrane (Merck Millipore Isopore; 0.1 µm pore size; Merck HGaA, Darmstadt, Germany) placed 45 mm from the target. After deposition, oxygen was introduced into the chamber at a rate of 1 slm. More details on the deposition system are given in [27]. Figure 1 presents Transmission Electronic Microscopy (TEM) images of the nanopowders obtained in the two cases. Such TEM images were used to obtain the size distribution of the nanoparticles shown in Figure 2, which was well-fitted by a lognormal distribution. SAED (Selected Area Electron Diffraction) was used to identify the predominant composition of the NPs as Fe_3_O_4_, and their crystalline structure, as shown in Figure 3. The difference in nanoparticle dimensions was due both to the difference in pulse durations and repetition rate, which lead to a difference in interaction between the laser pulse and the ablated target and in expansion of the ablated target species between the target and the membrane on which they are collected [28,29]. The NPs which had a diameter of 50 nm were provided by NaBond Technologies Co, Ltd., Guangdong, China; the size distribution and XRD data are described in Reference [30].

The polymer used was commercially available polyethylenimine (PEI) (Sigma-Aldrich, Inc., Steinheim, Germany). The sensitive layer S1, consisting in polymer only, was obtained by mixing PEI with ethanol in a concentration of 5 mg/mL. The resulting solution was sprayed through a mask (with the role of protecting the IDTs) onto the quartz sensor substrate using an airbrush. Synthetic air was used as a carrier gas. In the case of sensitive layers S2–S6, after the corresponding mass of NPs was added to the 5 mg/mL polymer solution, the resulting suspension was sonicated for 15 min and deposited onto the substrate in the same way as S1. The thickness of the layers, as determined by WLI (White Light Interferometry), was about 250 nm.

**Figure 4:**
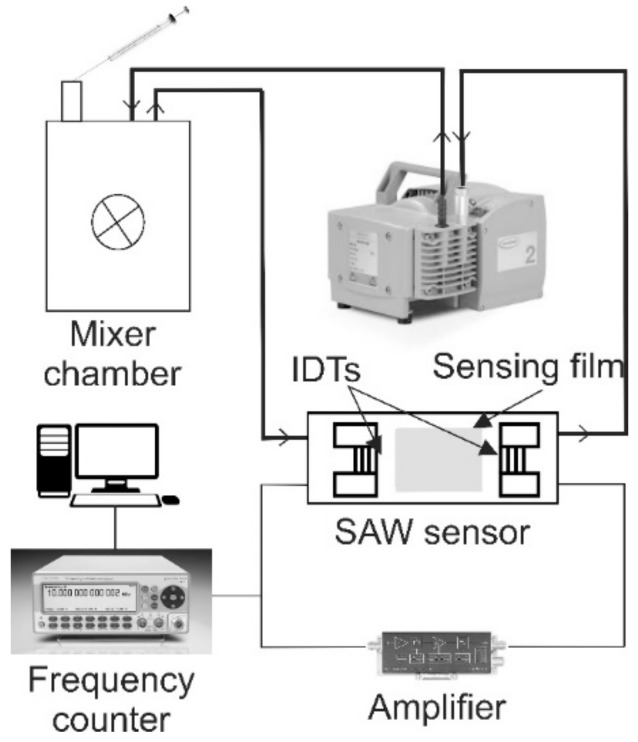
Experimental set-up used for measurements of sensor response to various Volatile Organic Compounds (VOCs).

A schematic diagram of the set-up used for sensor characterization is given in Figure 4. A CNT-91 Pendulum frequency counter, connected to a computer with Time View 3 software, was used to monitor the frequency change. A DHPVA-100 FEMTO amplifier (10–60 db, 100 MHz), was used to compensate the loss of the signal from the circuit. The response of the sensors to three different volatile organic compounds (VOCs) (ethanol, methanol and toluene) at concentrations between 160 and 16,000 ppm were determined. The acrylic mixer chamber has a volume of 8.5 L; calculated quantities of VOCs mixed with air were injected into it using a microliter pipette. The flow rate of the VOC/air mixture was maintained constant at 150 cm^3^/s with a diaphragm pump (Pfeiffer MVP 035-2) for all the measurements. The limit of detection (LOD) was defined as three times the noise level per sensitivity.

## 3. Results

**Figure 5:**
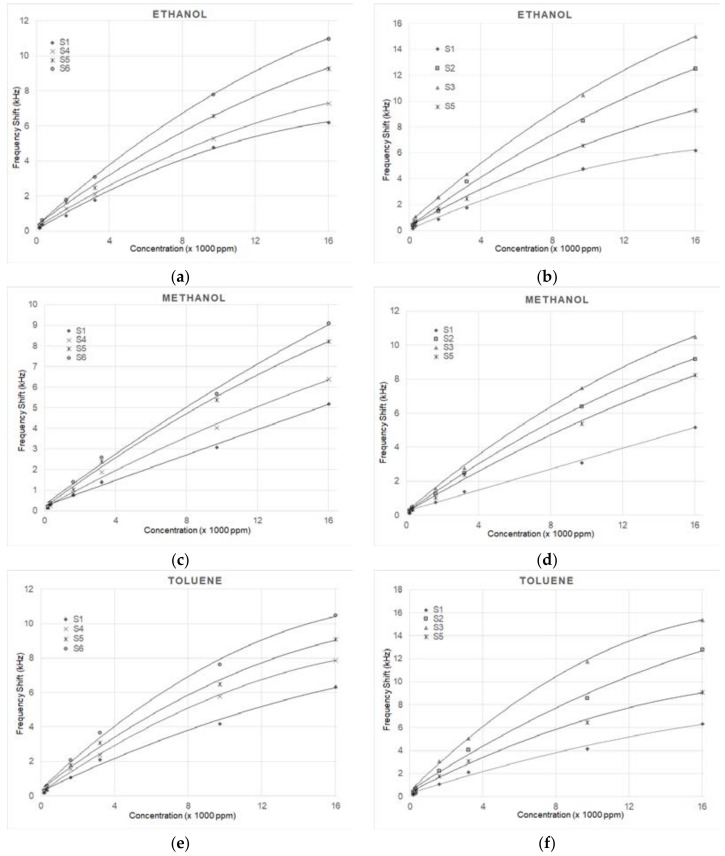
Frequency shift of the sensors for different concentrations of ethanol, methanol and toluene VOCs. (**a**,**c**,**e**) compare sensors all having 50 nm NPs and different NP concentrations, and sensor S1 with no NPs. (**b**,**d**,**f**) compare sensors with the same NP concentration of 0.4 mg/mL and different NP diameters, and sensor S1 with no NPs. See Table 1 for sensor identification.

Figure 5 gives the frequency shift of the sensors for the three VOCs at various concentrations. Figure 5a,c,e includes the results for the sensors having different concentrations of the same nanoparticle dimensions, while Figure 5b,d,f is for the same concentrations of different nanoparticle dimensions. All figures include the results for sample S1 with a polymer sensitive layer without NPs. As can be expected, the response increases for increasing concentrations. It is clear from all figures that the samples which contain NPs have a larger frequency shift than the one without NPs in all cases.

**Figure 6:**
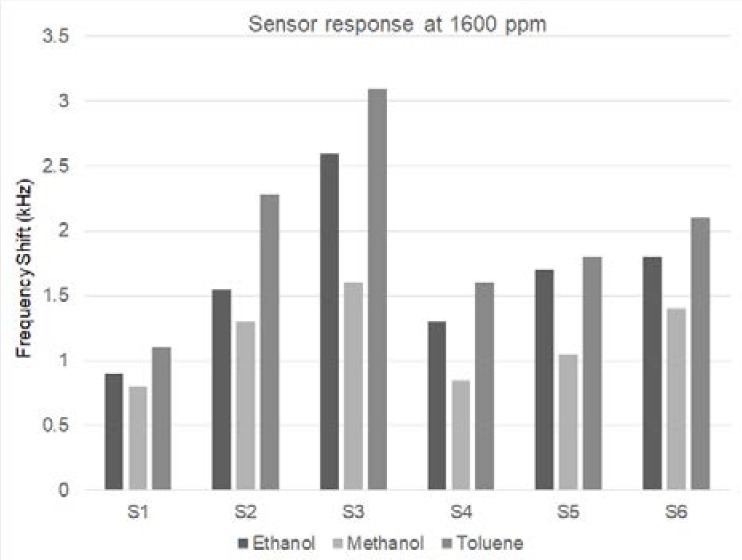
Frequency shift of sensors at 1600 ppm concentration of ethanol, methanol and toluene.

A comparison of the responses of the sensors which contain NPs is more visible in Figure 6, which gives the frequency shift of the 6 sensors to a concentration of 1600 ppm of the three VOCs. Sensor S3, which has the smallest nanoparticles, has the largest frequency shift for all three VOCs. Table 2 presents the sensitivities and LODs of the sensors for 1600 ppm of the VOCs. Here, again, it is evident that S3 has the best response, having the highest sensitivity and lowest LOD. On the other hand, the sensor without incorporated NPs has the lowest frequency shift and sensitivity, and highest LOD of all. It can be observed that the differences of sensitivity and LOD between different VOCs for the same sensor is significant, which proves the sensors’ selectivity. Repeating 10 measurements of the frequency deviation for each of the six sensors yielded errors below ±4%.

**Figure 7:**
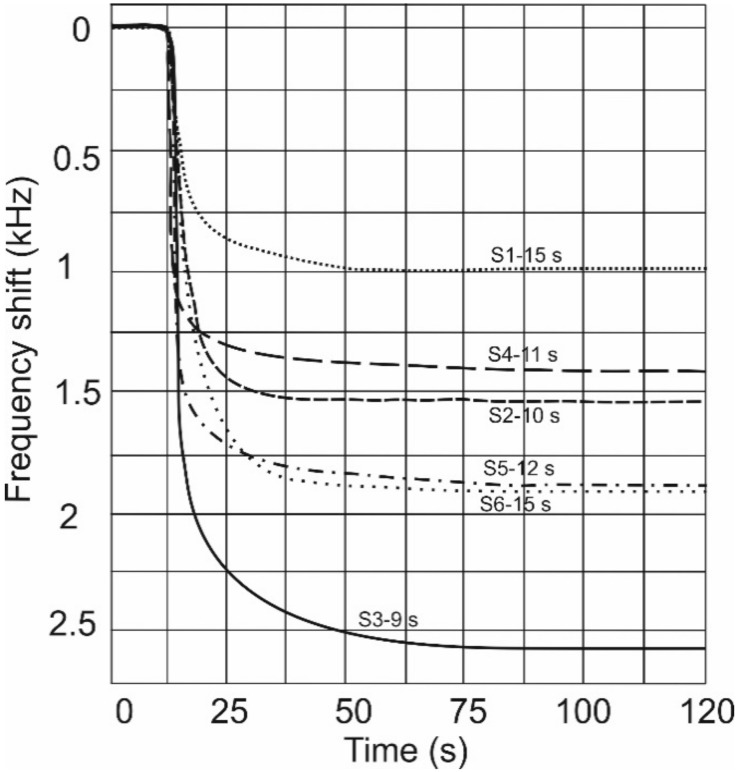
Response time of the devices at a concentration of 1600 ppm of ethanol.

The response time of the sensors studied was calculated as the necessary time for the oscillation frequency to reach 90% of maximum deviation signal from the frequency base line, and is presented in Figure 7.

## 4. Discussion and Conclusions

By comparing the sensors with NPs having different diameters and the same concentration, it is clear that the sensor with the smallest NP mean dimensions of 7 nm (and largest number of NPs) has the best properties, the largest frequency shift, and the highest sensitivity for all three VOCs studied. In addition, it has the lowest noise level of 35 Hz (which was determined from data collected for 10 min, and represents the maximum frequency deviation from the trend line) and thus the lowest limit of detection (LOD). The sensor with NPs having 13 nm mean dimensions has the next lowest LOD, and the highest frequency shift and sensitivity in the case of methanol and toluene.

Sensors with nanocomposite sensing films containing NPs with the same mean dimensions of 50 nm have frequency shifts and sensitivities which increase with NP concentration in the domain examined (0.2–0.8 mg/mL). However, the noise level also increases with NP concentration, reaching levels larger than in the case of the sensitive layer without NPs. The noise level for an NP concentration of 0.2 mg/mL (S4) is 55 Hz, while that for 0.8 mg/mL (S6) is 90 Hz; for the sensing layer with polymer only (S1) it is 60 Hz. Thus, the NP concentration cannot be increased indefinitely to improve sensor response.

As can be seen in Figure 7, the sensor with the 7 nm mean nanoparticle diameter has the shortest response time of about 9 s, while the one with a polymer only sensitive layer has the longest response time of about 15 s; both response times are for ethanol with 1600 ppm concentration. The differences between the response times of other sensors with incorporated NPs are not significant, so that no clear conclusions regarding them can be obtained. A possible explanation for a faster response of polymer films which contain NPs is that the structure of the nanocomposite films leads to a higher diffusivity of the VOCs [5]. In Reference [31], the solvent transport within a sensing layer consisting of NPs embedded in PEI was described by classical (Fickian) diffusion. The analysis allows us to conclude that the presence of NPs leads to an increase of the diffusion coefficient in the case of PEI, which in turn leads to a greater absorption speed of the sensing layer.

When a gas is adsorbed in the sensitive layer of the SAW sensor, a series of effects are produced and lead to a frequency shift. The main effects are mass loading, acoustoelectric loading and elastic loading [5,14]. There are several possible explanations for the improved sensor properties when NPs are incorporated into the polymer sensitive film. Introduction of NPs into the polymer increases the surface area, which in turn increases the sensor sensitivity [27]. Since preferred surface sites for gas adsorption are defects, it has been proven that smaller NPs lead to a larger number of sorption sites and sites at which gases react [23,32]. In the present case, for the same NP concentration, the total surface area of the NPs in sample S3 with 7 nm mean diameter is about twice that of sample S2 with 13 nm NPs and over seven times that of sample S5 with 50 nm NPs. This could explain the differences in these samples’ sensitivities; the only exception is the slightly smaller sensitivity of S2 compared to S5 for ethanol.

NPs also modify the electrical conductivity and elastic constant of the polymer matrix they are embedded in. The electrical conductivity of polymers with embedded NPs have been proven to increase for certain values of wt % NPs [33]. It has been established that an increase in the electron conductivity of polymers can enhance the sensitivity of SAW sensors through the increase in free charge carriers, which improves the coupling between the nanocomposite sensitive layer and the electric field associated with the SAW [10]. Research has also shown that incorporation of NPs into a polymer matrix leads to a linear increase of acoustic wave velocity and elastic constant with increasing NP concentration [20]. In particular, Fe_3_O_4_ NPs have been proven to increase the rigidity of a polymer, increase its viscosity and make an insulating polymer conductive even at low concentrations [34,35]. All these changes in the properties of the sensitive film lead to complex changes in its interaction with the gas and can potentially lead to improvement of the sensor performance.

NPs have also been proven to improve the uniformity of polymer sensitive films in certain conditions [5]. This in turn leads to a lower noise, since thickness variations of the film produce a loss of coherence of the propagating wavefront and a lower signal to noise ratio [15].

In conclusion, the properties of SAW sensors with polymer sensitive layers without embedded NPs and with NPs of different dimensions and concentrations have been analyzed. Their response (frequency shift, sensitivity, noise and response time) to three different VOCs at various concentrations were compared. The frequency shift and sensitivity increase with increasing NP concentration in the polymer for a given NP dimension and with decreasing NP diameter for a given concentration. The best results were obtained for the smallest NPs used, which led to the largest frequency shift and sensitivity, lowest noise and LOD and shortest response time for all VOCs. This SAW sensor containing 7 nm NPs had a limit of detection (LOD) of 65 ppm (almost five times better than the sensor with polymer alone), and a response time of about 9 s for ethanol. The clear dependence of SAW sensor performance on the dimensions of the NPs embedded in nanocomposite polymer sensitive layers has not, to our knowledge, been studied before.

## Figures and Tables

**Table 1:** Characteristics of nanocomposite sensitive layers of the sensors studied.

Sensor	Mean NP Diameter (nm)	NP Concentration (mg/mL)
S1	Polymer (PEI) only	0
S2	13	0.4
S3	7	0.4
S4	50	0.2
S5	50	0.4
S6	50	0.8

**Table 2:** Sensor characteristics for the three VOCs studied.

Sensor	Sensitivity (Hz/ppm)			LOD (ppm)		
	Ethanol	Methanol	Toluene	Ethanol	Methanol	Toluene
S1	0.56	0.50	0.69	320	360	262
S2	0.97	0.81	1.43	139	166	95
S3	1.63	1.00	1.94	65	105	54
S4	0.81	0.53	1.00	203	311	165
S5	1.06	0.66	1.13	212	343	200
S6	1.13	0.88	1.31	240	309	206

## References

[1] Devkota J., Ohodnicki P.R., Greve D.W. (2017). SAW Sensors for Chemical Vapors and Gases. Sensors.

[2] Hubert T., Boon-Brett L., Black G., Banach U. (2011). Hydrogen sensors—A review. Sens. Actuators B Chem..

[3] Buryakov I.A., Buryakov T.I., Matsaev V.T. (2014). Mass-Sensitive Micro- and Nanosensors for Detecting the Vapors of Explosives and Associated Substances. J. Anal. Chem..

[4] Viespe C. (2014). Surface Acoustic Wave Sensors based on Nanoporous Films for Hydrogen Detection. Materials and applications for sensors and transducers III. Key Eng. Mater..

[5] Yang L., Yin C., Zhang Z., Zhou J., Xu H. (2017). The investigation of hydrogen gas sensing properties of SAW gas sensor based on Pd surface modified SnO_2_ thin film. Mater. Sci. Semicond. Process..

[6] Ippolito S.J., Ponzoni A., Kalantar-Zadeh K., Wlodarski W., Comini E., Faglia G. (2006). Layered WO_3_/ZnO/36° LiTaO_3_ SAW gas sensor sensitive towards ethanol vapour and humidity. Sens. Actuators B Chem..

[7] Jakubik W.P. (2011). Surface acoustic wave-based gas sensors. Thin Solid Films.

[8] Viespe C., Miu D. (2017). Surface Acoustic Wave Sensor with Pd/ZnO bilayer structure for room temperature hydrogen detection. Sensors.

[9] Marcu A., Nicolae I., Viespe C. (2016). Active surface geometrical control of noise in nanowire-SAW sensors. Sens. Actuators B Chem..

[10] Penza M., Tagliente M.A., Aversa P., Cassano G. (2005). Organic-vapor detection using carbon-nanotubes nanocomposite microacoustic sensors. Chem. Phys. Lett..

[11] Al-Mashat L., Tran H.D., Wlodarski W., Kaner R.B., Kalanter-zadeh K. (2008). Polypyrrole nanofiber surface acoustic wave gas sensors. Sens. Actuators B Chem..

[12] Joo B.-S., Huh J.-S., Lee D.-D. (2007). Fabrication of polymer SAW sensor array to classify chemical warfare agents. Sens. Actuators B Chem..

[13] Horrillo M.C., Fernandez M.J., Fontecha J.L., Sagayo I., Garcia M., Aleixandre M., Santos J.P., Ares L., Gutierrez J., Garcia I. (2004). Detection of volatile organic compounds using surface acoustic wave sensors with different polymer coatings. Thin Solid Films.

[14] Matatagui D., Marti J., Fernandez M.J., Fontecha J.L., Gutierrez J., Gracia I., Cane C., Horillo M.C. (2009). Optimized design of a SAW sensor array for chemical warfare agents simulants detection. Procedia Chem..

[15] Ballantine D.S., White R.M., Martin S.I., Ricco A.J., Zellers E.T., Frye G.C., Wohltjen H. (1997). Acoustic Wave Sensors, Theory, Design and Physico-Chemical Applications.

[16] Dinca V., Fardel R., Shaw-Stewart F., Di Pietrantonio D., Cannata M., Benetti E., Verona A., Palla-Papavlu A., Dinescu M., Lippert T. (2010). Laser-induced forward transfer: An approach to single-step polymer microsensor fabrication. Sens. Lett..

[17] Dinca V., Palla-Papavlu A., Dinescu M., Shaw-Stewart F., Lippert T., Di Pietrantonio D., Cannata M., Benetti E., Verona A. (2010). Polymer pixel enhancement by laser-induced forward transfer for sensor applications. Appl. Phys. A.

[18] Wei D.W., Wang L.S., Ma J.Y., Jiang H.M. (2012). Synthesis and evaluation of hexafluoroisopropanol-functionalized polysiloxane as a new coating material for sensors. J. Appl. Polym. Sci..

[19] Viespe C., Grigoriu C. (2010). Surface acoustic wave sensors with carbon nanotubes and SiO_2_/Si nanoparticles based nanocomposites for VOC detection. Sens. Actuators B Chem..

[20] Kim H., Choi Y.-J., Kang K.-M., Park H.-H. (2014). Directly patternable SnO_2_ thin films incorporating Pt nanoparticles. Mater. Res. Bull..

[21] Kaushik A., Kumar R., Arya S.K., Nair M., Malhotra B.D., Bhansali S. (2015). Organic-Inorganic Hybrid Nanocomposite-Based Gas Sensors for Environmental Monitoring. Chem. Rev..

[22] Iqbal S., Ahmad S. (2018). Recent developments in hybrid conducting polymers: Synthesis, applications and future prospects. J. Ind. Eng. Chem..

[23] Su P.G., Peng Y.-T. (2014). Fabrication of a room-temperature H_2_S gas sensor based on PPy/WO_3_ nanocomposite films by in-situ photopolimerization. Sens. Actuators B Chem..

[24] Fu C., Lee K.J., Yang S.S. (2014). Low intensity ultraviolet detection using a surface-acoustic-wave sensor with a Ag-doped ZnO nanoparticle films. Smart Mater. Struct..

[25] Nicolae I., Viespe C., Grigoriu C. (2011). Nanocomposite sensitive polymeric films for SAW sensors deposited by the MAPLE direct write technique. Sens. Actuators B Chem..

[26] Holmes M.A., Mackay M.E., Giunta R.K. (2007). Nanoparticles for dewetting suppression of thin polymer films used in chemical sensors. J. Nanopart. Res..

[27] Viespe C., Grigoriu C. (2013). SAW sensor based on highly sensitive nanoporous palladium thin film for hydrogen detection. Microelectron. Eng..

[28] Huotari J., Kekkonen V., Haapalainen T., Leidinger M., Sauerwald T., Puustinen J., Liimatainen J., Lappalainen J. (2016). Pulsed laser deposition of metal nanostructures for highly sensitive gas sensor applications. Sens. Actuators B Chem..

[29] Makarov G.N. (2013). Laser Applications in nanotechnology: Nanofabrication using laser ablation and laser nanolithography. Physics-Uspekhi.

[30] Radu M., Dinu D., Sima C., Burlacu R., Hermenean A., Ardelean A., Dinischiotu A. (2015). Magnetite nanoparticles induced adaptive mechanisms countered cell death in human pulmonary fibroblasts. Toxicol. In Vitro.

[31] Nicolae I., Viespe C., Serban N., Negrila C.C., Teodorescu V.S., Trupina L. (2013). Increased Diffusion Coefficient of Polymeric Nanocomposite Layer for Gas Sensing Applications. Sens. Lett..

[32] Dharmelingam G., Joy N.A., Grisafe B., Carpenter M.A. (2012). Plasmonics-based detection of H_2_ and CO: Discrimination between reducing gases facilitated by material control. Beilstein J. Nanotechnol..

[33] Van S.N., Hadji R., Vincent B., Rouxel D., Sarry F., Bauer F. P(VDF-TrFE)/Al_2_O_3_ piezoelectric thin films. Proceedings of the 2010 IEEE International Symposium on the Applications of Ferroelectrics (ISAF).

[34] Essabir H., Raji M., Essassi E.M., Rodrigue D., Bouhfid R., Qaiss A. (2017). Morphological, thermal, mechanical, electrical and magnetic properties of ABS/PA6/SBR blends with Fe_3_O_4_ nano-particles. J. Mater. Sci. Mater. Electron..

[35] Weidenfelder B., Hoefer M., Schilling F. (2002). Thermal and electrical properties of magnetite filled polymers. Compos. Part A Appl. Sci. Manuf..

